# Oestrogen receptor negative early operable primary breast cancer in older women—Biological characteristics and long-term clinical outcome

**DOI:** 10.1371/journal.pone.0188528

**Published:** 2017-12-28

**Authors:** Binafsha Manzoor Syed, DAL Morgan, Tulassi Setty, Andrew R. Green, Emma C. Paish, Ian O. Ellis, K. L. Cheung

**Affiliations:** 1 School of Medicine, University of Nottingham, Nottingham, United Kingdom; 2 Medical Research Centre, Liaquat University of Medical & Health Sciences, Jamshoro, Pakistan; 3 Department of Oncology, Nottingham University Hospitals, Nottingham, United Kingdom; University of North Carolina at Chapel Hill School of Medicine, UNITED STATES

## Abstract

**Background:**

Older women are at the greatest risk of breast cancer development and a considerable number present with comorbidities. Although the majority of breast cancers in this age group express oestrogen receptor (ER), which makes endocrine therapy (primary or adjuvant) feasible, given the huge size of the elderly population, there remains a significant number of patients, in absolute term, whose tumours do not express ER and their management is challenging.

**Methods:**

Of a consecutive series of 1,758 older (≥70 years) women with early operable primary breast cancer managed in a dedicated service from 1973–2010, 252(14.3%) had ER-negative (histochemical (H) score ≤50) tumours. Their clinical outcome was retrospectively reviewed and tumour samples collected from diagnostic core biopsies were analysed for progesterone receptor (PgR), HER2 and Ki67 using immunohistochemistry.

**Results:**

The commonest primary treatment was surgery (N = 194, 77%) followed by primary endocrine therapy (14.3%), primary radiotherapy (5.6%) and supportive treatment only (3.1%). Among the patients undergoing surgery, most of them had grade 3 (78.1%) and node-negative disease (62.2%). Some of them (21.1%) received postoperative radiotherapy. At a median follow-up of 37.5 months, 117 patients had died, out of which 48.6% were due to breast cancer. For those who underwent surgery, the regional and local recurrence rates were 2% and 1.1% per annum respectively. For those who received primary endocrine therapy, 38% progressed at 6 months, however all patients who had primary radiotherapy achieved clinical benefit at 6 months. Regardless of treatment given, the 5-year breast cancer specific and overall survival rates were 70% and 50% respectively. Biological analysis based on good quality needle core biopsy specimensfrom181 patients showed that 26.8% (N = 49), 16.9% (N = 31) and 70.7% (N = 70)expressed positivity for PgR, HER2 and Ki67 respectively. No correlation between these biomarkers and breast cancer specific survival was demonstrated.

**Conclusion:**

Oestrogen receptor negative early operable primary breast cancer in older women is associated with poor prognostic features in terms of biology and clinical outcome. Surgery appears to produce the best outcome as a primary treatment, however for those where neither surgery nor chemotherapy is appropriate, primary radiotherapy can be beneficial.

## Introduction

The risk of developing breast cancer rises with advancing age and one-third of cases occur in women over 70 years of age[1]. The highest life time probability of breast cancer development was observed in women ≥70 years of age (6.73%) as compared to their younger counterparts where the probability reported was 1.44%, 2.39% and 3.40% respectively in the age groups of <40, 40–50 and 51–60 years[2]. Despite this, most research efforts are directed towards younger patients. The currently available literature showsa remarkable lack or under-representation of older women in clinical trials.

With increasing age there is evidence available showing a rise in oestrogen receptor (ER) positivity, where over 80% of older (≥70 years) women tend to develop ER-positive tumours[3], which are also considered as a less aggressive phenotype. However, given the size of the elderly population, there still remain a significant number of women, in absolute terms, who develop ER-negative tumours with possible poor prognosis. On the other hand, older women show a high prevalence of comorbidities, where a considerable proportion are life-threatening [4]. Patients with ER-positive tumours have endocrine therapy (primary or adjuvant) as a viable option. However, those with ER-negative tumours still pose a therapeutic challenge to clinicians. Partly because of their relatively small number (relative to their ER-positive counterparts within the elderly cohort), little is known about their biological characteristics and long-term clinical outcome.

A UK-based audit of management protocols in older women with early operable primary breast cancer reported that 40% of them were treated by non-operative therapy[5]. This fact has impacted on how ER-negative primary breast cancer in older women could be studied. Most of the available studies only include patients treated by surgery (often using surgical specimens for studying biological features), introducing a potential bias towards a comparatively fitter older population. If we were to encompass all older women with ER-negative tumours regardless of primary treatment, we have to study their biological features using tumour tissue obtained from diagnostic needle core biopsies. The limitations of such approach include the small quantity and quality of the specimen, and method used to process the tissue (eg fresh frozen or formalin fixed paraffin embedded tissues).

This study aimed to evaluate a series of ER-negative tumours from a large consecutive cohort of older women with early operable primary breast cancer, regardless of their initial primary treatment, by analysing (1) their long-term clinical outcome; and (2) their biological features in terms of progesterone receptor (PgR), human epidermal receptor (HER) 2 and Ki67.

## Patients and methods

The study involved 252 older (≥70 years of age) women with ER-negative early operable invasive primary breast carcinoma (defined as T0-2, N0-1, M0) diagnosed between 1973 and 2010. Data analysis using cut-offs of ER histochemical (H)-score of 0 and 50 (using immunohistochemistry (IHC)) to define ER negativity showed no significant difference in clinical outcome, thus for the purpose of this paper ER negativity was defined as H-score ≤50. H-score ≤50 has been reported to have fairly low expression merely predicting endocrine response[6]. These patients were identified from a well characterised consecutive series of 1,758 (out of which ER status was available in 1,380 patients) older women managed in a dedicated service for early operable primary breast cancer at Nottingham University Hospitals, as previously described in details[7, 8]. Briefly, patients were identified from a database in Histopathology Department (using paper archive (before 1987) and computerised records recently). Clinical data were then retrospectively collected from clinical notes. Basic histopathological characteristics i.e. types, grades and ER status were based on standard reports of diagnostic needle core biopsies by a single team of pathologists following the same pathological guidelines at any time point. Axillary stage was defined according to the number of involved lymph nodes in the surgical specimen (therefore only applicable to patients treated by primary surgery): 1 = 0 involved, 2 = 1–3 involved and 3 = ≥4 involved[9].

There have been changes in the management guidelines over the period concerned, as previously described[7], however most of these changes were attributed to the use of endocrine therapy in clinical practice, having minimal impact on the ER-negative group. Thus their general management approach essentially remained the same throughout, though in the recent decade the dedicated clinic had evolved to become a joint surgical/oncology facility, supported by dedicated breast care nurses, with more structured multidisciplinary assessment. Regardless, all patients were offered surgery as primary therapy with axillary surgery as appropriate (before year 2000 axillary surgery was only considered when there were clinically node positive disease, while from 2000,level III axillary clearance was carried out for pre-operatively proven node-positive cases), and four-node sampling, with introduction of blue dye-assisted sentinel node biopsy in recent years, was performed in clinically node-negative cases. In the recent decade, intact breast irradiation was given to all patients following wide local excision (WLE) and post-mastectomy chest wall irradiation was given if two of the following three features existed—grade 3, positive lymph nodes or vascular invasion. Adjuvant chemotherapy with/without trastuzumab was recommended to comparatively fit patients based on their prognostic features and HER2 status. Those who were unfit or refused to have surgery were then assessed and offered primary radiotherapy as appropriate, using a variety of dose schedules and techniques, all intended to deliver the biological equivalent of at least 70Gy in 35 fractions to the site of the primary tumour, with lower doses to remaining ipsilateral breast tissue. Historically some patients received primary endocrine therapy (using tamoxifen or an aromatase inhibitor such as anastrozole if they had contraindications to or were unable to tolerate tamoxifen). All patients were followed up on a regular basis except for those who were frail and/or refused further follow-up, they were discharged back to the general practitioner (GP)’s care.

The outcome of all patients was analysed in terms of breast cancer specific survival and overall survival, defined respectively as the interval from diagnosis to death from breast cancer, and as the interval from diagnosis to death from any causes. Those treated by primary surgery were assessed for time to local and regional recurrences, defined as the time from surgery to appearance of the first local (in the intact breast for patients undergoing WLE, and in the anterior chest wall for patients treated by mastectomy, all on the ipsilateral side) or regional (in the ipsilateral axilla) recurrence respectively. Response to non-operative therapy was assessed clinically by callipers, according to International Union Against Cancer (UICC) criteria and British Breast group recommendations [10, 11]. Complete response (CR) was defined as complete disappearance of tumour, partial response (PR) as >50% reduction, stable disease (SD) as <50% reduction or <25% increase, progressive disease (PD) as >25% increase in the bi-dimensional product of the tumour. Appearance of a new tumour (including metastases) was also categorised as PD. Clinical benefit (CB) was defined by CR/PR/SD for a minimum duration of 6 months[12].

### Biological analysis of needle core biopsies

Out of 252 patients with ER-negative tumours, 181 sets of good quality needle core biopsy specimens were available for biological analysis. Immunohistochemical (IHC) staining was performed specifically for this study, for PgR, HER2 and Ki67 by using StreptAvidin Biotin Complex (ABC) sequenza method, as previously described[13]. The description and the cut-off points to define positivity of the markers are given in Table 1.

**Table 1:** Summary of immunohistochemistry methodology for biological analysis.

Primary antibody	Source	Dilution	Incubation time	Cut-off to define positivity
PgR	Dako- Mouse monoclonal	1:100	45 minutes	≥1%
HER2	Dako-Rabbit and anti-human	1:250	45 minutes	3+
Ki67	Dako- MIB1 clone	1:300	45 minutes	≥10%

### Statistical methods

Statistical analysis was carried out using Statistical Package for Social Sciences (SPSS, version 17, Chicago, Illinois). A p-value of <0.05 was considered significant. Time-dependant variables were analysed by using Kaplan-Meier method with application of Log-rank and Wilcoxon tests of significance as appropriate.

### Additional information required

Oestrogen receptor (ER) negative breast cancers in older women pose a clinical dilemma, where the majority presents with a number of co-morbidities and related social issues. Currently there is limited literature available to guide in this regard. This study presents biological characteristics and clinical outcome in a large series with ER negative breast cancers in older women.

#### Ethics statement

The study was approved by the Research Ethics Committee at Nottingham City Hospital. The requirement for informed consent was waived.

## Results

### All patients

#### Patients’ characteristics and management pattern

As described above, a total of 252 patients were found to have ER negative tumours with H-score ≤50, with the great majority having no ER expression at all (H-score 0 = 90.5%, N = 228, H-score 1–50 = 9.5%, N = 24). Out of these 71% were <80 years of age. The majority of them had grade 2 or3 tumours(45.4% and 50.0% respectively) and invasive ductal carcinoma with no special type (NST) was the most common histological type observed on needle core biopsy specimens (Table 2).

**Table 2:** Patient and tumour characteristics and management pattern of ER-negative early operable primary breast cancer in older women.

	**Number of patients**	**Percentage (%)**
**Age (years)**	**N = 252**	
70–79	179	71
More than 80	73	29
**Clinical size of tumour (cm)**	**N = 247**	
0(Screen detected)	14	5.7
0.1–2	57	23.0
2.1–5	176	71.3
**Treatment groups**	**N = 252**	
Surgery	194	77
Primary endocrine therapy	36	14.3
Primary radiotherapy	14	5.6
No treatment (supportive care only)	8	3.1
**Pattern of surgical management**	**N = 194**	
Mastectomy	155	79.7
WLE*	39	20.3
Axillary surgery	135	69.6
**Adjuvant Radiotherapy**		
Adjuvant radiotherapy	41	29.9
**Grade (based on core biopsies)**	**N = 108**	
1	5	4.6
2	49	45.4
3	54	50.0
**Grade (based on surgical specimen)**	**N = 174**	
1	5	2.9
2	33	19.0
3	136	78.1
**Axillary stage**	**N = 135**	
1	84	62.2
2	29	21.5
3	22	16.3
**Histological types (based on core biopsies)**	**N = 193**	
Ductal carcinoma (including NST**)	162	84.2
Tubular mixed	5	2.5
Lobular	5	2.5
Mucinous	1	0.5
Other types	20	10.3
**Biomarkers**		
**PgR**	**N = 183**	
Positive	49	26.8
Negative	134	73.2
**HER2**	**N = 183**	
Positive	31	16.9
Negative	152	83.1
**Ki67**	**N = 99**	
Positive	70	70.7
Negative	29	29.3
**Outcome**	**Overall rate (%)**	**Average rate per annum (%)**
**Local recurrence**		
All	10.4	2.0
Mastectomy group	8.4	1.0
WLE*	18.4	1.8
**Regional recurrence**		
All	8.3	1.1
Axillary surgery	5.9	0.9
No axillary surgery	13.8	1.8

*Wide local excision,

**No special type

Most (N = 194, 77%) patients underwent surgery as their primary treatment (mastectomy = 79.7%, WLE = 20.3%), with axillary surgery in 69.9% of them. The second most common primary treatment was primary endocrine therapy (N = 36, 14.3%), followed by primary radiotherapy (N = 14, 5.6%). Eight (3.1%) patients did not have any active treatment and received supportive care only.

#### Causes of death and survival analysis

**Fig 1:**
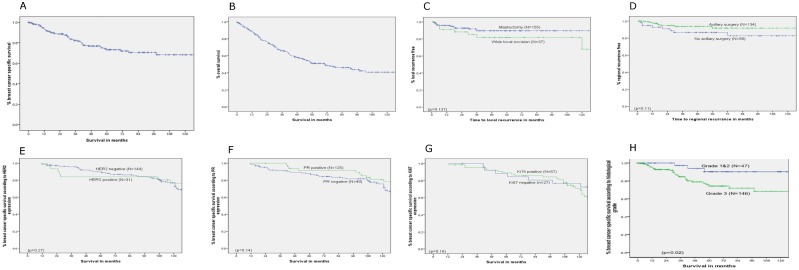
Kaplan-Meier plots of time-dependant outcome variables in older women with ER-negative early operable primary breast cancer. (A) Breast cancer specific survival of Oestrogen receptor negative early operable primary breast cancer in older women. (B) Overall survival of Oestrogen receptor negative early operable primary breast cancer in older women. (C) Local recurrence free survival of Oestrogen receptor negative early operable primary breast cancer in older women- Mastectomy versus wide local excision. (D) Regional recurrence free survival of Oestrogen receptor negative early operable primary breast cancer in older women- Axillary surgery versus no axillary surgery. (E) Breast cancer specific survival of Oestrogen receptor negative early operable primary breast cancer in older women- Progesterone Receptor (PR) positive versus negative. (F) Breast cancer specific survival of Oestrogen receptor negative early operable primary breast cancer in older women- Human Epidermal Growth Factor Receptor– 2 (HER2) positive versus negative. (G) Breast cancer specific survival of Oestrogen receptor negative early operable primary breast cancer in older women- Ki67 positive versus negative. (H) Breast cancer specific survival of Oestrogen receptor negative early operable primary breast cancer in older women- Low (ie 1–2) Grade versus high (ie 3) Grade.

At a median follow-up of 37.5 (longest 220) months, 107 patients had died, of which 48.6% (N = 52) were from breast cancer (Table 3). A relatively higher proportion of patients diagnosed at the age of <80 years died of breast cancer (54.5% breast cancer deaths versus 45.5% non-breast cancer deaths), as compared to the older patients (33.3% breast cancer deaths versus 66.7% non-breast cancer deaths). Among the non-breast cancer deaths, a greater proportion (66.7% in ≥80 versus 45.5% in <80) were observed in the older (≥80 years) age group (p = 0.03). The 5-year breast cancer specific survival and overall survival rates were 70% and 50% respectively (Fig 1).

**Table 3:** Causes of death by age groups in older women with ER-negative early operable primary breast cancer.

Age groups	Breast cancer deaths N (%)	Non-breast cancer deaths N (%)	Total	p-value
All	52 (48.6)	55 (51.4)	107	0.08
70–79 years	42 (54.5)	35 (45.5)	77	0.42
≥80 years	10 (33.3)	20 (66.7)	30	0.06

### Patients treated by surgery

#### Management pattern and clinical outcome

For those patients who underwent surgery (N = 194),the majority had grade 3 as confirmed on operative histology (78.2%) and for those who had axillary surgery, most of them (62.2%) had node-negative disease. Forty-one patients (29.9%) had adjuvant radiotherapy and none of them received adjuvant chemotherapy.

After a median follow-up of 37.5 months,75 (38.7%) patients died, out of which 33 died from breast cancer and the rest from non-breast cancer related causes. The 5-year breast cancer survival and overall survival rates were 80% and 61% respectively. The local and regional recurrence rates were 2% and 1.1% per annum respectively (Table 2). There was no significant difference between the rates of and time to local recurrence, regardless of whether they underwent mastectomy or WLE. There was no significant difference in regional recurrence between patients who had axillary surgery and those who did not (Fig 1).

#### Patients treated by non operative therapy

Fifty patients received non-operative therapy including primary endocrine therapy (N = 36) and primary radiotherapy (N = 14).

#### Primary endocrine therapy

A total of 36 patients were put on primary endocrine therapy; 27 (75.0%) had tumours with H-score 0 and the rest had H-score between 1–49. Response status at 6 months was available in 26 patients, of which 15 (57.7%) achieved CB, 10 (38.5%) progressed and one patient (3.8%) came off treatment due to intolerance. Eventually 25 patients progressed after a median duration of 6 months (longest 45 months). For those who achieved CB, the median duration was 17 (range 9–45) months.

At a median follow-up of 23 (longest 103) months, 33 (91.7%) patients died. Among those whose causes of death were known, 15 and 13 died of breast cancer and non-breast cancer causes respectively.

#### Primary radiotherapy

A total of 14 patients received primary radiotherapy. After a median follow-up of 15.5 months nine patients were still alive, three died from non-breast cancer causes (two of them died within 6 months) and two patients died of breast cancer. Within 6 months two patients died without progression and two were discharged back to GP’s care thus the response status at 6 months was available in 10 patients. All of them achieved CB (CR = 50%, PR = 40%, SD = 10%). At the time of analysis only three patients progressed respectively at 9, 33 and 34 months (median time to progression = 33 months). The median duration of CB was 34 months (longest 52 months). A summary of all patients, including their survival, is given in Table 4.

**Table 4:** Summary of patients with ER-negative early operable primary breast cancer treated by primary radiotherapy.

Patient number	Age (years)	ER H-score	Response at 6 months	Progression status	Time to progression (months)	Second line Therapy	Survival (months)	Survival status at last follow-up
1	70	0	CR	No	-	-	14	Alive
2	70	0	CR	No	-	-	52	Alive
3	73	0	PR^2^	No	-	-	9	Died from non BC
4	74	0	CR	No	-	-	25	Alive
5	75	0	NA^3^	NA	-	-	30	Died from BC
6	79	0	PR	Yes	9	Mastectomy	11	Alive
7	79	0	CR^1^	Yes	33	Tamoxifen	166	Died from BC^4^
8	79	0	SD	No	-	-	9	Alive
9	80	0	NA	Na	-	-	5	Died from non BC
10	81	0	NA	NA	-	-	4	Died from non BC
11	81	0	NA	NA	-	-	1	Alive
12	82	0	PR	Yes	34	No treatment	69	Alive
13	85	20	PR	No	-	-	17	Alive
14	91	0	CR	No	-	-	26	Alive

^1^ = Complete response,

^2^ = Partial response,

^3^ = Not available*,

^4^ = Breast cancer

#### Biological analysis

As described, 185 patients had additional IHC analysis of further biomarkers based on core biopsy specimens. Positive expression of PgR, HER2 and Ki67 was observed respectively in 49 (26.8%), 31 (16.9%) and 70 (69.8%) patients. A summary of the data is given in Table 2. None of the three markers showed significant influence on breast cancer specific survival (Fig 1).

## Discussion

Results of the analysis showed that the majority of older women with ER-negative early operable primary breast cancer presented with larger tumour size, and high grade, ductal carcinomas. A great majority of them had primary surgery and only a small number was treated by non-operative therapies. A considerable proportion died of breast cancer. For those treated by primary surgery, the local and regional failure rates were low (approximately 2% and 1% per annum respectively). For patients treated by non-operative means, the response to primary endocrine therapy was poor, while primary radiotherapy was associated with a relatively better outcome, However given the small number of patients, it would be difficult to draw a conclusion.

Regardless of treatment pattern and biological markers, the absence of ER expression has been shown to be associated with poor prognosis[8, 14]. The degree of ER positivity has been reported in younger (<70 years) patients as a significant indicator of outcome[6]. Patients who had tumours with poor ER expression (ie having a low H-score) showed poorer outcome following surgery and adjuvant tamoxifen without any chemotherapy, when compared to those with a higher ER H-score[6]. As described, therapeutic options for ER-negative tumours in the elderly population, especially those with significant comorbidities, are limited. Individual randomised trials or even meta-analysis of large number of trials have not yet been able to provide sufficient evidence for wide recommendation of chemotherapy in older women. However the Oxford Overview showed that the number of patients >70 years available for meta-analysis was limited[15].

In this series the majority of patients were treated by surgery with adjuvant radiotherapy as appropriate but no adjuvant chemotherapy. Non-operative therapy, as reported by other studies, is usually prescribed due to patients’ choice, ill health or in some cases extreme of age which make surgery an inappropriate option[5, 16]. However to date very limited data are available reporting on the role of non-operative therapy in this group of patients. In contrast to the outcome of patients with ER-positive tumours as reported by our group previously (from the large cohort of patients managed in the same dedicated service), where the majority died of non breast cancer causes[8], the majority of those with ER-negative tumours studied here died of breast cancer. The annual rates of local and regional recurrences were nearly double when compared with that seen in ER positive tumours and also the survival outcome was relatively poor (Table 5).

**Table 5:** Clinical outcome and expression of biomarkers in ER-negative versus ER-positive tumours in older women with early operable primary breast cancer.

Age group	ER negative (present study)		ER positive(8)	
	Breast cancer deaths N(%)	Non-breast cancer deaths N(%)	Breast cancer deaths N(%)	Non-breast cancer deaths N(%)
All	52 (48.6)	55(51.4)	105 (25.8)	302 (74.2)
70–79 years	42 (54.5)	35(45.4)	70 (34.7)	132 (65.3)
≥80 years	10 (33.3)	20(66.7)	35 (17.1)	170 (82.9)
Expression of biomarkers				
Biomarker	ER negative (present study)		ER positive [17]	
PR positive (%)	26.8		46	
HER2 positive (%)	16.9		8	
Ki67 positive (%)	70.7		64	

While this could indicate the aggressive tumour biology of ER-negative tumours, on the other hand, it may suggest sub-optimal treatment. Not every patient in the series was able to have surgery which was shown to be associated with the best outcome. Endocrine therapy does not play any role in ER-negative tumours. High level evidence is scarce in supporting the use of cytotoxic chemotherapy with or without trastuzumab in the elderly population.

In contrast to what was seen by our group in the ER-positive disease, the higher rates of Ki67 expression, relatively lower proportion of PR expression and higher proportion of HER2 over-expression (as opposed to the ER positive tumours) in our study (Table 5)are consistent with more aggressive tumour biology as reported in previous studies[14, 17–20].

With advancing age biological characteristics of breast cancer appear to change with high rate of ER positivity, PR positivity and low HER2 and Ki67 expression in general[3], however the extent of these changes is not yet clear in ER-negative tumours. Further studies in this regard are strongly recommended. Those patients with HER2 over-expressing tumours have trastuzumab as a potential therapeutic option. However available evidence has primarily been drawn from studies focusing on younger patients, with no or under-representation of older women and invariably all studies demonstrated the role of trastuzumab given in combination with cytotoxic chemotherapy[21–23]. The presence of comorbidities, limited physiological reserve and lack of social support may make chemotherapy very challenging in this group of patients and trastuzumab mono-therapy could be very attractive though there is no evidence to support its use at present.

The response to primary endocrine therapy appeared poor in general as compared to surgery, which is not unexpected due to the relatively low ER expression and PgR expression (26.8%). However the use of primary endocrine therapy in this series was predominantly historical (1970s and early 1980s mainly). Although primary radiotherapy has produced a comparatively better outcome, it would be difficult to draw a definite conclusion given the small patient number. Nevertheless it could be considered as a therapeutic option for patients with very limited life expectancy and when surgery is deemed inappropriate. Although there is limited data available showing the role of primary radiotherapy for early operable primary breast cancer which is normally treated by primary surgery, it has been used in locally advanced breast cancer[24]. Further studies in the future may be able to delineate its precise role in this selected group of elderly patients. Two studies reported on hypo-fractionated radiotherapy (single weekly dose of 6.5 Gy corresponding to 5 fractions, with total dose of 32.5–45.5 Gy) in combination with tamoxifen 20 mg in older women, given as primary therapy because they refused surgery for their newly diagnosed primary breast cancer [25, 26]. Both studies included patients of 64 years and older with T1-4, N0-1, M0 disease with a sample size of 70 (78% ER positive) in one study and 115 (68% ER positive) in the other. The study by Maher et al, at a median follow-up of 36 months, reported 10 deaths, out of which eight were due to breast cancer, giving a 3-year disease specific survival rate of 88%. Eleven patients developed loco-regional failure and another 11 developed distant metastases, leaving 72% of patients disease free at the time of analysis [25]. The study by Courdi et al, at 41 months’ median follow-up, reported 15% (N = 19) local failure and 78% 5-year local progression free survival, while the 5-year breast cancer specific survival rate was 71% [26]. Given that tamoxifen was used alongside radiotherapy in both studies, it would be difficult to extract the precise effect of primary radiotherapy. Regardless there is a possible role of radiotherapy (in full dose or in hypofractionation form) in this context, but a definitive recommendation is not possible with such limited data.

In conclusion, older women with ER negative early operable primary breast cancer appeared to have poor prognosis, however those treated by surgery or primary radiotherapy had relatively better outcome, while primary endocrine therapy was associated with poor outcome. Regardless of treatment the majority of patients died of breast cancer. The results of this study strongly recommend consideration of primary surgery in most patients if comorbidities allow, and further studies to explore the precise role of chemotherapy and trastuzumab,. Other less toxic biological therapies should also be explored as potential therapeutic options. Primary radiotherapy may also be used as an alternative to surgery in selected cases. Hypofractionated schedules of radiotherapy, entailing far fewer hospital visits than previously given, are much more attractive for these patients, and increasing hypofractionation (use of even fewer, but larger individual fractions) is currently being evaluated in many centres.

## Supporting information

S1 File Table A, Age Pattern. Table B, Clinical Size of tumours. Table C, Treatment groups. Table D, Progesterone receptor (PR) status. Table E, Human Epidermal Growth Factor Receptor (HER)2 status. Table F, KI67 status. Table G, Clinical Outcome. Table H, Causes of Death. Fig A & B, Clinical outcome.
(DOCX)
