# Correction: Oestrogen receptor negative early operable primary breast cancer in older women—Biological characteristics and long-term clinical outcome

**DOI:** 10.1371/journal.pone.0191979

**Published:** 2018-01-25

**Authors:** Binafsha Manzoor Syed, DAL Morgan, Tulassi Setty, Andrew R. Green, Emma C. Paish, Ian O. Ellis, K. L. Cheung

The following information is missing from the Funding section: This study is part of a research program funded by Breast Cancer Research Trust, UK.
