# Supplementary material for: Oestrogen receptor negative early operable primary breast cancer in older women—Biological characteristics and long-term clinical outcome
Source: PLoS One. 2017 Dec 28;12(12):e0188528. doi: 10.1371/journal.pone.0188528 (PMC5746234; doi:10.1371/journal.pone.0188528)
Supplement: S1 File — Table A, Age Pattern. Table B, Clinical Size of tumours. Table C, Treatment groups. Table D, Progesterone receptor (PR) status. Table E, Human Epidermal Growth Factor Receptor (HER)2 status. Table F, KI67 status. Table G, Clinical Outcome. Table H, Causes of Death. Fig A & B, Clinical outcome. (DOCX) [file pone.0188528.s001.docx]

Table A: Age Pattern

| **Age at presentation** | | | | | |
| --- | --- | --- | --- | --- | --- |
|  |  | Frequency | Percent | Valid Percent | Cumulative Percent |
| Valid | 70 | 16 | 6.3 | 6.3 | 6.3 |
|  | 71 | 10 | 4.0 | 4.0 | 10.3 |
|  | 72 | 18 | 7.1 | 7.1 | 17.5 |
|  | 73 | 25 | 9.9 | 9.9 | 27.4 |
|  | 74 | 20 | 7.9 | 7.9 | 35.3 |
|  | 75 | 24 | 9.5 | 9.5 | 44.8 |
|  | 76 | 24 | 9.5 | 9.5 | 54.4 |
|  | 77 | 20 | 7.9 | 7.9 | 62.3 |
|  | 78 | 8 | 3.2 | 3.2 | 65.5 |
|  | 79 | 14 | 5.6 | 5.6 | 71.0 |
|  | 80 | 9 | 3.6 | 3.6 | 74.6 |
|  | 81 | 10 | 4.0 | 4.0 | 78.6 |
|  | 82 | 9 | 3.6 | 3.6 | 82.1 |
|  | 83 | 7 | 2.8 | 2.8 | 84.9 |
|  | 84 | 6 | 2.4 | 2.4 | 87.3 |
|  | 85 | 6 | 2.4 | 2.4 | 89.7 |
|  | 86 | 7 | 2.8 | 2.8 | 92.5 |
|  | 87 | 2 | .8 | .8 | 93.3 |
|  | 88 | 5 | 2.0 | 2.0 | 95.2 |
|  | 89 | 3 | 1.2 | 1.2 | 96.4 |
|  | 90 | 5 | 2.0 | 2.0 | 98.4 |
|  | 91 | 3 | 1.2 | 1.2 | 99.6 |
|  | 92 | 1 | .4 | .4 | 100.0 |
|  | Total | 252 | 100.0 | 100.0 |  |

| **agegroups** | | | | | |
| --- | --- | --- | --- | --- | --- |
|  |  | Frequency | Percent | Valid Percent | Cumulative Percent |
| Valid | 70-79 | 179 | 71.0 | 71.0 | 71.0 |
|  | =>80 | 73 | 29.0 | 29.0 | 100.0 |
|  | Total | 252 | 100.0 | 100.0 |  |

Table B : Clinical Size of tumours

| **clinical size in 3 groups with 0** | | | | | |
| --- | --- | --- | --- | --- | --- |
|  |  | Frequency | Percent | Valid Percent | Cumulative Percent |
| Valid | 0 | 14 | 5.6 | 5.7 | 5.7 |
|  | 0.1-2.0 | 57 | 22.6 | 23.1 | 28.7 |
|  | 2.1-5 | 176 | 69.8 | 71.3 | 100.0 |
|  | Total | 247 | 98.0 | 100.0 |  |
| Missing | System | 5 | 2.0 |  |  |
| Total | | 252 | 100.0 |  |  |

Table C. Treatment groups

Table C1. Treatment groups

| **Treatment recode 1** | | | | | |
| --- | --- | --- | --- | --- | --- |
|  |  | Frequency | Percent | Valid Percent | Cumulative Percent |
| Valid | No treatment | 8 | 3.2 | 3.2 | 3.2 |
|  | PET | 36 | 14.3 | 14.3 | 17.5 |
|  | Surgery | 194 | 77.0 | 77.0 | 94.4 |
|  | Radiotherapy | 14 | 5.6 | 5.6 | 100.0 |
|  | Total | 252 | 100.0 | 100.0 |  |

Table C2. Treatment groups

| **recode of treatment with surgery 2 groups** | | | | | |
| --- | --- | --- | --- | --- | --- |
|  |  | Frequency | Percent | Valid Percent | Cumulative Percent |
| Valid | Mastectomy | 155 | 79.9 | 79.9 | 79.9 |
|  | WLE | 39 | 20.1 | 20.1 | 100.0 |
|  | Total | 194 | 100.0 | 100.0 |  |

Table C3. Treatment groups

| **axillary surgery groups** | | | | | |
| --- | --- | --- | --- | --- | --- |
|  |  | Frequency | Percent | Valid Percent | Cumulative Percent |
| Valid | No axillary surgery | 59 | 30.4 | 30.4 | 30.4 |
|  | axillary surgery | 135 | 69.6 | 69.6 | 100.0 |
|  | Total | 194 | 100.0 | 100.0 |  |

Table C4. Treatment groups

Radiotherapy after surgery

| **RT after initial surgical rx** | | | | | |
| --- | --- | --- | --- | --- | --- |
|  |  | Frequency | Percent | Valid Percent | Cumulative Percent |
| Valid | No | 104 | 41.3 | 71.7 | 71.7 |
|  | Yes | 41 | 16.3 | 28.3 | 100.0 |
|  | Total | 145 | 57.5 | 100.0 |  |
| Missing | System | 107 | 42.5 |  |  |
| Total | | 252 | 100.0 |  |  |

Table D. Progesterone receptor (PR) status

| **PgR_posneg_y** | | | | | |
| --- | --- | --- | --- | --- | --- |
|  |  | Frequency | Percent | Valid Percent | Cumulative Percent |
| Valid | 0 | 134 | 72.4 | 73.2 | 73.2 |
|  | 1 | 49 | 26.5 | 26.8 | 100.0 |
|  | Total | 183 | 98.9 | 100.0 |  |
| Missing | System | 2 | 1.1 |  |  |
| Total | | 185 | 100.0 |  |  |

Table E . Human Epidermal Growth Factor Receptor (HER)2 status

| **HER2_posneg_y** | | | | | |
| --- | --- | --- | --- | --- | --- |
|  |  | Frequency | Percent | Valid Percent | Cumulative Percent |
| Valid | 0 | 152 | 82.2 | 83.1 | 83.1 |
|  | 1 | 31 | 16.8 | 16.9 | 100.0 |
|  | Total | 183 | 98.9 | 100.0 |  |
| Missing | System | 2 | 1.1 |  |  |
| Total | | 185 | 100.0 |  |  |

Table F . KI67 status

| **Ki67_posneg_y** | | | | | |
| --- | --- | --- | --- | --- | --- |
|  |  | Frequency | Percent | Valid Percent | Cumulative Percent |
| Valid | 0 | 29 | 15.7 | 29.3 | 29.3 |
|  | 1 | 70 | 37.8 | 70.7 | 100.0 |
|  | Total | 99 | 53.5 | 100.0 |  |
| Missing | System | 86 | 46.5 |  |  |
| Total | | 185 | 100.0 |  |  |

Tables G . Clinical Outcome

Table G1. Clinical Outcome

Local recurrence

| **Local recurrence status** | | | | | |
| --- | --- | --- | --- | --- | --- |
|  |  | Frequency | Percent | Valid Percent | Cumulative Percent |
| Valid | no | 173 | 89.2 | 89.6 | 89.6 |
|  | yes | 20 | 10.3 | 10.4 | 100.0 |
|  | Total | 193 | 99.5 | 100.0 |  |
| Missing | System | 1 | .5 |  |  |
| Total | | 194 | 100.0 |  |  |

Table G2. Clinical Outcome

| **Case Processing Summary** | | | | |
| --- | --- | --- | --- | --- |
| recode of treatment with surgery 2 groups | Total N | N of Events | Censored | |
|  |  |  | N | Percent |
| Mastectomy | 155 | 13 | 142 | 91.6% |
| WLE | 37 | 7 | 30 | 81.1% |
| Overall | 192 | 20 | 172 | 89.6% |


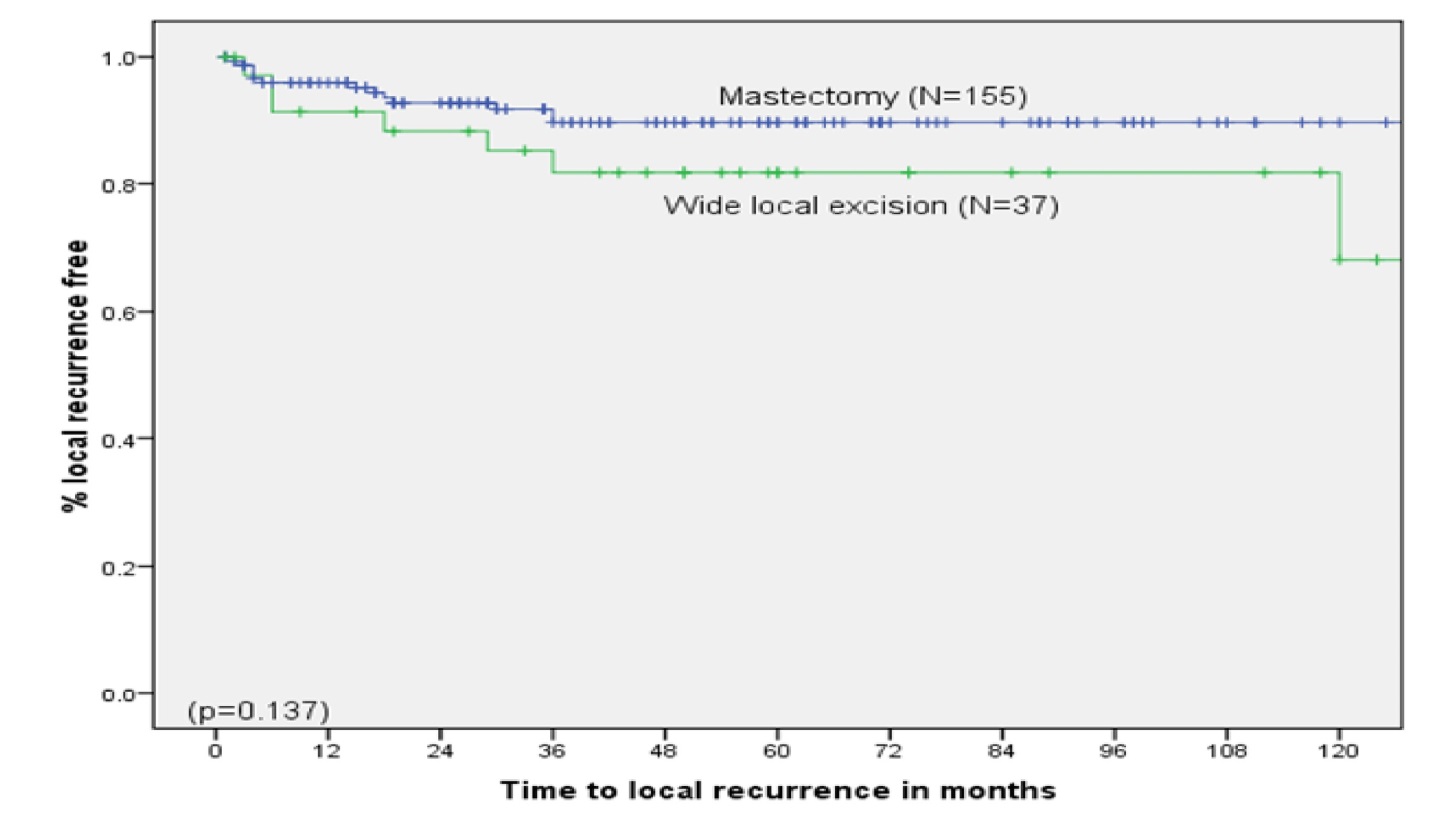


Figure A. Local recurrence free survival

Table G2. Clinical Outcome

Regional recurrence

| **Regional recurrence status** | | | | | |
| --- | --- | --- | --- | --- | --- |
|  |  | Frequency | Percent | Valid Percent | Cumulative Percent |
| Valid | no | 177 | 91.2 | 91.7 | 91.7 |
|  | yes | 16 | 8.2 | 8.3 | 100.0 |
|  | Total | 193 | 99.5 | 100.0 |  |
| Missing | System | 1 | .5 |  |  |
| Total | | 194 | 100.0 |  |  |

| **Case Processing Summary** | | | |
| --- | --- | --- | --- |
| Total N | N of Events | Censored | |
|  |  | N | Percent |
| 192 | 16 | 176 | 91.7% |

TableG3. Clinical Outcome

| **Case Processing Summary** | | | | |
| --- | --- | --- | --- | --- |
| axillary surgery groups | Total N | N of Events | Censored | |
|  |  |  | N | Percent |
| No axillary surgery | 58 | 8 | 50 | 86.2% |
| axillary surgery | 134 | 8 | 126 | 94.0% |
| Overall | 192 | 16 | 176 | 91.7% |


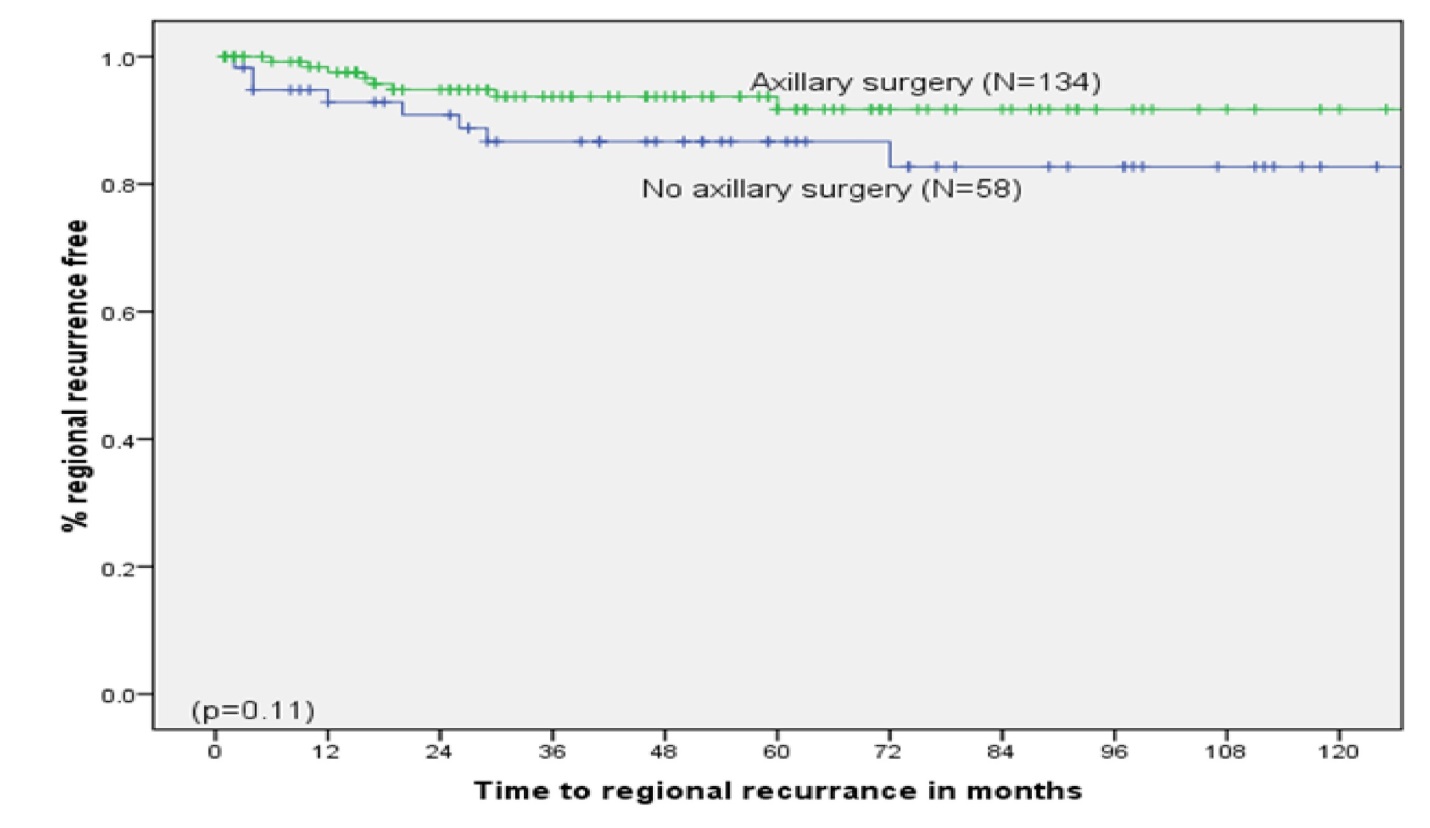


Fig B . Regional recurrence free survival

Table H1. Causes of Death

| **Is the Patient Dead** | | | | | |
| --- | --- | --- | --- | --- | --- |
|  |  | Frequency | Percent | Valid Percent | Cumulative Percent |
| Valid | Dead from disease | 52 | 48.6 | 48.6 | 48.6 |
|  | Dead from other causes | 55 | 51.4 | 51.4 | 100.0 |
|  | Total | 107 | 100.0 | 100.0 |  |

Table H2. Causes of Death

| **Is the Patient Dead * agegroups Crosstabulation** | | | | | |
| --- | --- | --- | --- | --- | --- |
|  |  |  | agegroups | | Total |
|  |  |  | 70-79 | =>80 |  |
| Is the Patient Dead | Dead from disease | Count | 42 | 10 | 52 |
|  |  | % within Is the Patient Dead | 80.8% | 19.2% | 100.0% |
|  |  | % within agegroups | 54.5% | 33.3% | 48.6% |
|  | Dead from other causes | Count | 35 | 20 | 55 |
|  |  | % within Is the Patient Dead | 63.6% | 36.4% | 100.0% |
|  |  | % within agegroups | 45.5% | 66.7% | 51.4% |
| Total | | Count | 77 | 30 | 107 |
|  |  | % within Is the Patient Dead | 72.0% | 28.0% | 100.0% |
|  |  | % within agegroups | 100.0% | 100.0% | 100.0% |
